# THE INTERSECTION OF VULNERABILITY AND OLD AGE: ETHICAL CONSIDERATIONS FOR LONG-TERM SERVICES AND SUPPORTS

**DOI:** 10.1093/geroni/igz038.890

**Published:** 2019-11-08

**Authors:** Kelly Niles-Yokum

**Affiliations:** University of La Verne, La Verne, California, United States

## Abstract

This session focuses on ethical considerations in the context of long-term services and supports (LTSS) for vulnerable older adults. Long-term supports for vulnerable older adults can no longer adhere to a “one-sized fits all” solution. We will explore the intersection of vulnerability, old age, and community which present a myriad of ethical issues in both the planning and delivery of supports for older adults. The quest for a just society goes beyond understanding and considering the critical issues of the vulnerability of older adults in our society in that this pursuit provides a pathway to develop and implement programs and services that allow all of us the opportunity to live in a world that both protects and can provide the opportunity for self determination and dignity.

